# Glutamate/GABA+ ratio is associated with the psychosocial domain of autistic and schizotypal traits

**DOI:** 10.1371/journal.pone.0181961

**Published:** 2017-07-31

**Authors:** Talitha C. Ford, Richard Nibbs, David P. Crewther

**Affiliations:** 1 Centre for Human Psychopharmacology, Faculty of Heath, Arts and Design, Swinburne University of Technology, Melbourne, Victoria, Australia; 2 Swinburne Neuroimaging, Faculty of Heath, Arts and Design, Swinburne University of Technology, Melbourne, Victoria, Australia; University of Minnesota Twin Cities, UNITED STATES

## Abstract

**Background:**

The autism and schizophrenia spectra overlap to a large degree in the social and interpersonal domains. Similarly, abnormal excitatory glutamate and inhibitory *γ*-aminobutyric acid (GABA) neurotransmitter concentrations have been reported for both spectra, with the interplay of these neurotransmitters important for cortical excitation to inhibition regulation. This study investigates whether these neurotransmitter abnormalities are specific to the shared symptomatology, and whether the degree of abnormality increases with increasing symptom severity. Hence, the relationship between the glutamate/GABA ratio and autism and schizophrenia spectrum traits in an unmedicated, subclinical population was investigated.

**Methods:**

A total of 37 adults (19 female, 18 male) aged 18-38 years completed the Autism Spectrum Quotient (AQ) and Schizotypal Personality Questionnaire (SPQ), and participated in the resting state proton magnetic resonance spectroscopy study in which sequences specific for quantification of glutamate and GABA+ concentration were applied to a right and left superior temporal voxel.

**Results:**

There were significant, moderate, positive relationships between right superior temporal glutamate/GABA+ ratio and AQ, SPQ and AQ+SPQ total scores (p<0.05), SPQ subscales Social Anxiety, No Close Friend, Constricted Affect, Odd Behaviour, Odd Speech, Ideas of Reference and Suspiciousness, and AQ subscales Social Skills, Communication and Attention Switching (p<0.05); increased glutamate/GABA+ coinciding with higher scores on these subscales. Only the relationships between glutamate/GABA+ ratio and Social Anxiety, Constricted Affect, Social Skills and Communication survived multiple comparison correction (p< 0.004). Left superior temporal glutamate/GABA+ ratio reduced with increasing restricted imagination (p<0.05).

**Conclusion:**

These findings demonstrate evidence for an association between excitatory/inhibitory neurotransmitter concentrations and symptoms that are shared between the autism and schizophrenia spectra.

## Introduction

Symptoms within many psychiatric disorders exist on a spectrum from clinical pathology to subclinical personality traits. Autism and schizophrenia are among these spectrum disorders, with trait phenotypes reliably identified in the high functioning population using psychometric instruments such as the Autism Spectrum Quotient (AQ [1]) and the Schizotypal Personality Questionnaire (SPQ [2]). Subscales within these instruments were intended to reflect specific symptoms of the respective disorder, insofar as social and communication deficits, and restricted and repetitive interests and behaviours are measured by the AQ, and the positive, negative and disorganized dimensions of schizophrenia spectrum disorders are measured by the SPQ.

The general consensus amongst the literature is that autism and schizophrenia spectrum disorders overlap to a large degree at a clinical [3–5] and subclinical level [6–9], particularly in the psychosocial domain. Specifically, higher scores on the AQ subscales Social Skills and Communication have been closely related to higher scores on the SPQ subscales Constricted Affect, Social Anxiety and No Close Friends [3, 5–7], as well as the disorganized subscales [10]. This overlap questions the capacity of the AQ and the SPQ to discriminate between similarities across the two spectra, and instead indicates that a shared symptom phenotype exists.

Through factor analysis of the AQ and SPQ subscales, a phenotype with major contributions from the AQ subscales Social Skills, Communication and Attention Switching, and SPQ subscales No Close Friends, Constricted Affect, Social Anxiety, Odd Behaviour and Odd Speech was revealed [7]. This factor was termed Social Disorganization [7], supporting Dinsdale et al.’s [6] first principal component of general social-communicative disinterest, impairments and abnormalities. As autism and schizophrenia are largely idiopathic, heterogeneous disorders with complex neural underpinnings, investigating specific phenotypes within the disorders is therefore necessary to identify phenotype neural correlates. This is a necessary step in the development of more specific diagnostic practices and treatment interventions.

Recently, findings from our lab have demonstrated differences in neurotransmitter concentrations between high and low Social Disorganization scorers, using proton magnetic resonance spectroscopy (^1^H-MRS). These include a higher excitatory glutamate to inhibitory *γ*-aminobutyric acid (GABA) ratio in the right superior temporal lobe for those with a greater degree of the Social Disorganization phenotype was revealed [11]. Abnormal levels of ^1^H-MRS quantified glutamate and GABA have been previously reported in autism and schizophrenia [12–19]. Taken together, these results suggest that the glutamate/GABA ratio might be associated with a trait phenotype that is shared across the autism and schizophrenia spectra.

Glutamate is synthesised from glutamine via phosphate-activated glutaminase in glutamatergic neurons and astrocytes [20, 21]. Glutamate plays a central role in excitatory neurotransmission, excitotoxicity, cortical metabolism, and the mediation of GABA inhibitory neurotransmission [20]. Glutamate is also essential for neuroplasticity—for synaptic reorganisation, as well as for cellular differentiation, migration and cell death [22]. As a mediator of excitatory signalling, glutamate is involved in cognition, learning and memory [22].

The neurotransmitter GABA is synthesised via glutamic acid decarboxylase (GAD) from glutamate [20], and regulates glutamatergic excitation in the synapse. Concurrently, the neurotransmitter glutamate stimulates GABAergic interneurons by binding to N-methyl-D-aspartate receptors (NMDArs), which increases the excitatory postsynaptic potential (EPSP) within the interneuron, and thus promotes inhibition [23, 24]. An imbalance in the ratio of excitatory to inhibitory neurotransmitters, particularly excess excitation, has been associated with cortical instability and compromised plasticity, which subsequently affects learning, cognition and behaviour [25, 26]. The right balance of glutamate/GABA is therefore essential for normal cortical functioning [25, 26].

Several studies have demonstrated relationships separately between autism and schizophrenia spectrum disorders on the one hand, and glutamate and GABA concentrations on the other [12–14, 16–18]. Negative symptoms, social and communication deficits, and poorer global functioning have been shown to increase with more anterior cingulate cortex (ACC) glutamate/creatine [16, 27], and temporal lobe glutamate+glutamine (Glx) concentration [28]. AQ scores have also been shown to increase with left auditory glutamate concentration [13] and psychosocial dysfunction has been associated with increased excitation/inhibition ratio [29]. Finally, increased severity of autistic symptoms and social cognitive dysfunction have been related to reduced GABA/creatine ratio in the ACC [30, 31].

This study investigates the association between the glutamate/GABA ratio and the AQ and SPQ subscales, particularly those within the Social Disorganization phenotype. It was hypothesised that increased glutamate/GABA ratio in a right superior temporal voxel would be associated with higher scores on the AQ subscales Social Skills, Communication and Attention Switching, and SPQ subscales No Close Friends, Constricted Affect, Social Anxiety, Odd Behaviour and Odd Speech.

## Materials and methods

### Participants

The Swinburne University Human Research Ethics Committee approved this study, and all participants provided written informed consent prior to commencement. These methods have been reported in a manuscript that is under review elsewhere. A total of 37 adults (19 female, 18 male, age 18-38) participated in this study. Demographic information is presented in Table 1. No participants reported a current psychiatric illness although five reported a personal psychiatric history (3 depression, 1 bipolar, 1 anorexia). All participants were free of psychiatric medication, illicit drug and nicotine effects at the time of the ^1^H-MRS scan.

**Table 1:** Imaging participant demographics (mean(SD)).

	Female	Male
n	19	18
Age	22.47(4.61)	23.78(5.96)
Social Dis. (z score)	-0.09(1.53)	0.25(1.49)
AQ (/200)	111.53(21.97)	114.17(20.55)
SPQ (/296)	145.79(43.51)	152.78(41.98)
RAPM-S (/12)	7.42(2.09)	6.44(2.25)

Social Dis. = Social Disorganization, AQ = Autism Spectrum Quotient, SPQ = Schizotypal Personality Questionnaire, RAPM-S = Raven’s Advanced Progressive Matrices–Short form.

### Autism schizotypy questionnaire (ASQ)

Participants completed the ASQ on-line using the Opinio software interface [32]. The ASQ included all questions from the AQ, SPQ, Coolidge Axis II Inventory (CATI+) Schizotypy and Schizoid scale, and short Eysenck Personality Questionnaire Revised Lie scale (EPQR-L) items. All items were presented on a 4-point Likert scale from 1(*strongly disagree*) to 4(*strongly agree*).

The AQ consists of 50-items, yielding a total score out of 200, that measure autistic tendency through five subscales: Social Skills, Communication, Attention Switching, Attention to Detail and Imagination [1]. The SPQ consists of 74-items, yielding a total score out of 296, that measure schizotypal personality traits across the three dimensions of the schizophrenia spectrum; these dimensions are encapsulated in nine subscales: Ideas of Reference, Odd Beliefs, Unusual Perceptual Experiences, and Suspiciousness (Cognitive-Perceptual features); Social Anxiety, No Close Friends and Constricted Affect (Interpersonal features); Odd Behaviour and Odd Speech (Disorganized features) [2]. The CATI+ Schizotypy subscale measures degree of odd beliefs and magical thinking, unusual perceptual experiences, suspiciousness, inappropriate or odd behaviour, and social anxiety through 22 items [33]. The CATI+ Schizoid scale measures long term absence of social relationships, solitary activities, muted affect and withdrawal on 10 items. Finally, the EPQR-L assesses social desirability and response honesty through 12 items [34].

Intelligence was measured with the Raven’s Advanced Progressive Matrices–Short form (RAPM-S), consisting of 13 items from the original test [35]. Participants were given ten minutes to complete the RAPM-S and correct answers were summed to give a total out of 13.

### ^1^H-MRS protocol

**Fig 1:**
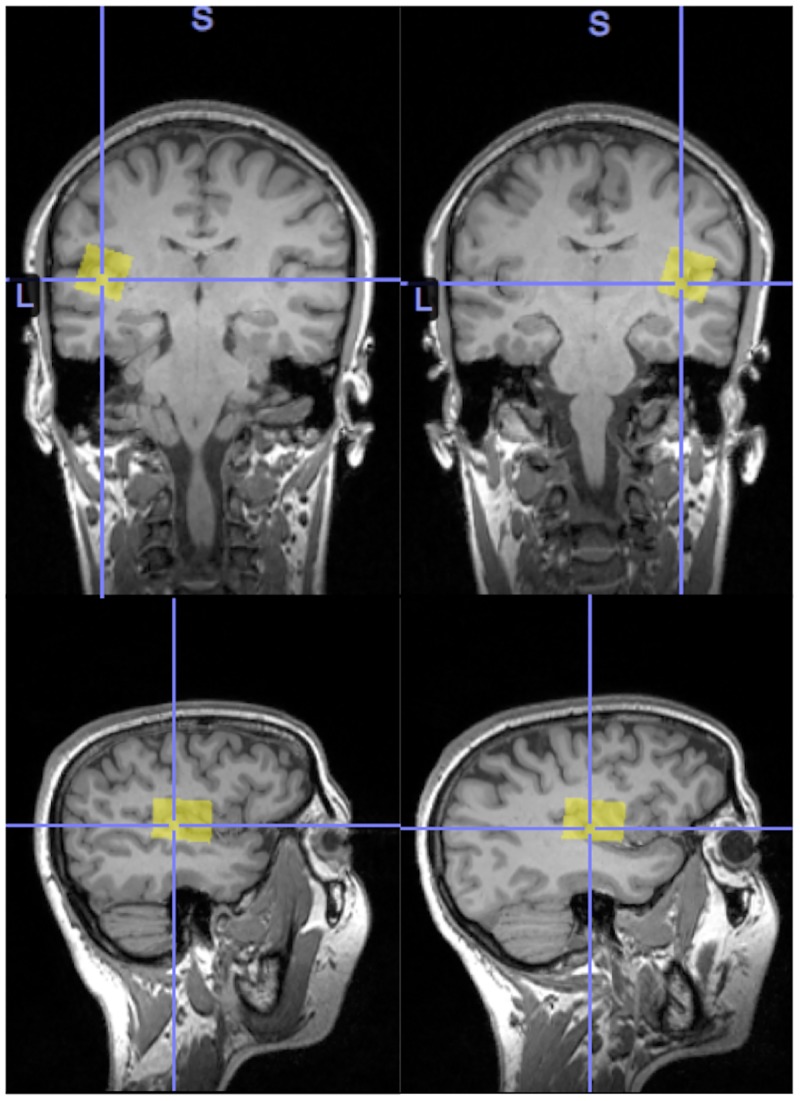
Example temporal lobe ^1^H-MRS voxel placement. Example participant left (left panel) and right (right panel) superior temporal lobe voxel placement.

A 3T Siemens TIM Trio whole-body magnetic resonance imaging system (Erlangen, Germany) with a 32-channel head coil was used for ^1^H-MRS and T1-weighted image recordings. T1-weighted images were acquired for localisation of the left and right superior temporal lobe voxels (20x30x20mm, 176 slices, slice thickness = 1.0mm, voxel resolution = 1.0mm^3^, TR = 1900ms, TE = 2.52ms, TI = 900ms, bandwidth = 170Hz/Px, flip angle = 9°, field of view 350mm x 263mm x 350mm, orientation sagittal, acquisition time = 5min). See Fig 1 for example voxel placement.

Glutamate was quantified using an 80ms TE PRESS sequence to isolate the glutamate peak from the closely resonating glutamine peak [36] (TR = 2000ms, bandwidth = 1200Hz, 80 averages, acquisition time = 2 min 48 sec). Chemical shift selective (CHESS) water suppression was applied [37]. Eight spectral averages with water were acquired with identical PRESS parameters, except that water suppression was turned off.

Superior temporal GABA concentration was quantified using the MEGA-PRESS editing sequence [38, 39], whereby scans with a Gaussian editing pulse at 1.9ppm (edit-on) were interleaved with scans with a pulse at 7.5ppm (edit-off); CHESS water suppression was applied for all scans (TE = 68ms, TR = 1500ms, bandwidth = 1000Hz, edit pulse frequency = 1.9ppm, edit pulse bandwidth = 44Hz, 120 averages acquired, duration = 6min 6 sec). Twelve water spectra averages with identical MEGA-PRESS parameters were acquired with water suppression off.

**Fig 2:**
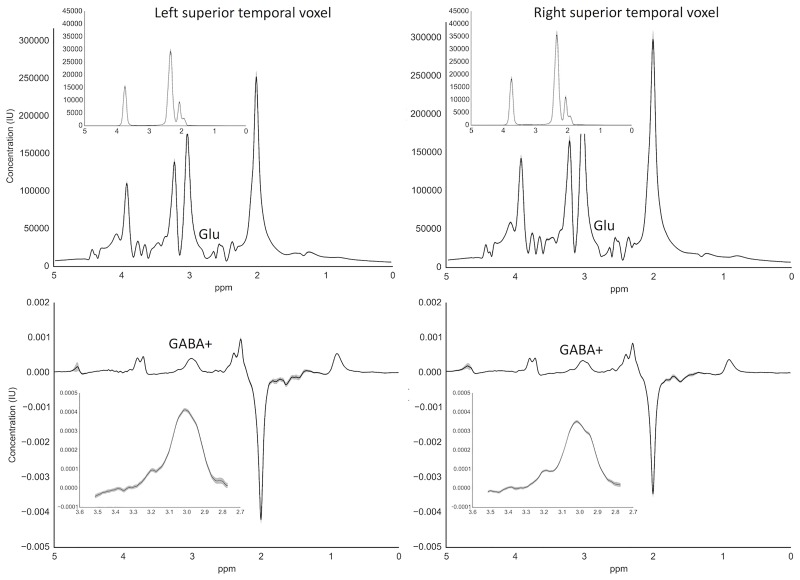
Average 80ms TE PRESS (for glutamate) and MEGA-PRESS spectra (for GABA+). Top: average left and right voxel 80ms TE PRESS spectra with isolated glutamate spectrum (inset) from TARQUIN. Bottom: average left and right voxel MEGA-PRESS edited spectra with isolated GABA+ peak at 3.0ppm (inset). The shading represents standard error from the mean.

Shimming was automatic and manual until the linewidth was less than 20Hz for the 80TE PRESS and MEGA-PRESS sequences. Fig 2 shows the average 80TE PRESS and MEGA-PRESS spectra from the left and right superior temporal voxel for all participants.

### Analysis

Glutamate concentration was obtained with TARQUIN version 4.3.7, which employs a non-negative least-squares projection of a parametrized basis set (alanine, aspartate, creatine, GABA, glucose, glutamine, glutamate, guanidinoacetate, myoinositol, lactate, N-acetyl-aspartate, N-acetyl-aspartylglutamate, scyllo-inositol, taurine, glycerophosphorylcholine and phosphorylcholine) to estimate signal amplitude in the time-domain [40]. Eddy current correction was applied to each participant spectrum. The fit of all participants glutamate spectra was adequate as shown in Table 2 and Fig 2, although one dataset was excluded due to large water drift (water frequency standard deviation = -6.88Hz). No right hemisphere data was recorded for one male participant.

**Table 2:** Tissue composition and 80TE PRESS (Glu) and MEGA-PRESS (GABA+) fit statistics by hemisphere.

	Left	Right
Gray matter	60.79(5.4)	63.3(6.13)
White atter	27.9(5.1)	24.83(6.13)
CSF	11.32(5.11)	11.87(4.04)
GABA+ Creatine FWHM	9.04(0.87)	9.04(0.8)
GABA+ water freq SD (Hz)	0.82(0.66)	1.08(1.1)
GABA+ fit error	10.6(2.66)	8.69(2.36)
GABA+ SNR	10.12(3.05)	12.31(3.17)
Glu water FWHM	7.62(1.07)	8.4(1.74)
Glu water freq SD (Hz)	0.42(1.56)	-0.21(2.44)
Glu CRLB	1.23(0.5)	0.99(0.61)
Glu SNR	45.94(9.95)	48.17(11.91)

CSF = cerebral spinal fluid, FWHM = full width half maximum, freq = frequency, SD = standard deviation, CRLB = Cramer-Rao lower bound, SNR = signal to noise ratio.

The Gannet GABA analysis toolkit for Matlab was used to analyse GABA data [41]. The edit-on and edit-off spectra are subtracted to reveal those molecules that are *J*-coupled to the peaks at 1.9ppm. The creatine peak is removed during the subtraction process. The edit-off pulse at 7.5ppm does not suppress macromolecules (MM) that are *J*-coupled to GABA at 3.0ppm, therefore the GABA concentration includes MMs and will subsequently be referred to as GABA+. Gannet models the GABA+ peak fit between 2.19ppm and 3.55ppm using a five-parameter Gaussian model. The water peak is modelled using a Gaussian-Lorentzian function. Gannet corrects for frequency and phase drift with spectral registration [42]. See Edden et al. [41] for a detailed description of the Gannet toolkit. All spectra had an adequate fit as shown in Table 2 and Fig 2.

As the concentration of GABA+ and glutamate in gray matter is twice that of white matter [43–45], it is currently best practice to correct for the proportion of gray matter per voxel, per participant. Gray matter correction was conducted using the equation given by Harris et al. [44], which also amounts to CSF correction [44]:

$$\begin{array}{c} c_{GMWMcorr}=\frac{c_{meas}}{\left(f_{GM}+\alpha f_{WM}\right)}\frac{\mu_{GM}+\alpha \mu_{WM}}{\mu_{GM}+\mu_{WM}} \end{array},$$

where *c*_*GMWMcorr*_ is the corrected metabolite concentration, *c*_*meds*_ is the original metabolite concentration, *f*_*GM*_ and *f*_*WM*_ are the individual’s GM and WM concentrations, and *μ*_*GM*_ and *μ*_*WM*_ are the group averaged gray matter and white matter concentrations [44]. *α* is the assumed ratio of the metabolite concentration in gray matter and white matter, and thus was set to 0.5 [43–45].

All descriptive and Spearman’s *ρ* rank-order correlation statistics were carried out using R. Spearman’s *ρ* rank-order correlations were calculated due to the non-normality of subscale scores. Kruskal-Wallis tests for non-normal distributions were reported when appropriate. Bonferroni correction for multiple comparisons (14 subscales) was applied (0.05/14 = 0.004).

## Results

There was no significant difference between males and females on total AQ (p = 0.69) or SPQ (p = 0.66) score, or performance on the RAPM-S measure of intelligence (p = 0.06), see Table 1.

**Fig 3:**
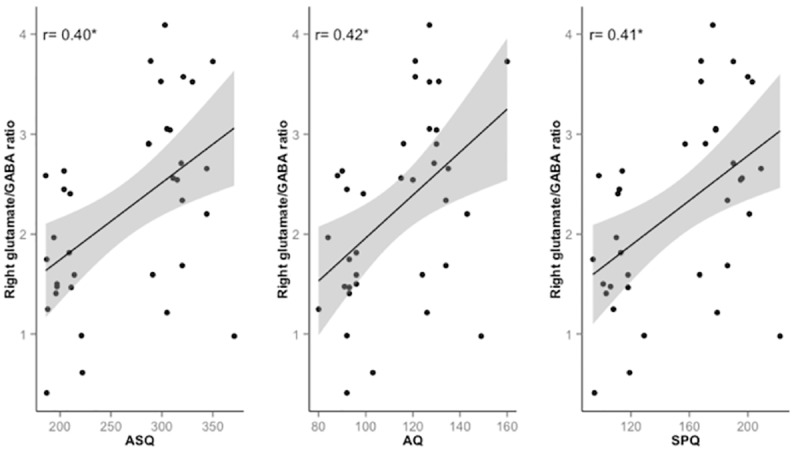
Significant Spearman’s *ρ* correlation coefficients between glutamate/GABA+ ratio and ASQ, AQ and SPQ scores. Scatter plots of right superior temporal voxel glutamate/GABA+ ratio versus total autism schizotypy questionnaire (ASQ), total autism spectrum quotient (AQ) and total schizotypal personality questionnaire (SPQ). *p< 0.05.

The Spearman’s *ρ* rank-order correlation coefficient and the coefficient of determination (adjusted *R*^2^) were calculated for the relationship between total AQ, SPQ and ASQ scores and glutamate/GABA+ ratio in right and left superior temporal voxel. There was a significant, moderate, positive correlation between right superior temporal glutamate/GABA+ ratio and AQ total (*ρ* = 0.42, p = 0.011), SPQ total (*ρ* = 0.41, p = 0.012) and ASQ total (*ρ* = 0.4, p = 0.014), with glutamate/GABA+ ratio increasing with higher total scores on each scale as shown in Fig 3. There were no significant left superior temporal glutamate/GABA+ relationships with total scales (all p> 0.05).

**Fig 4:**
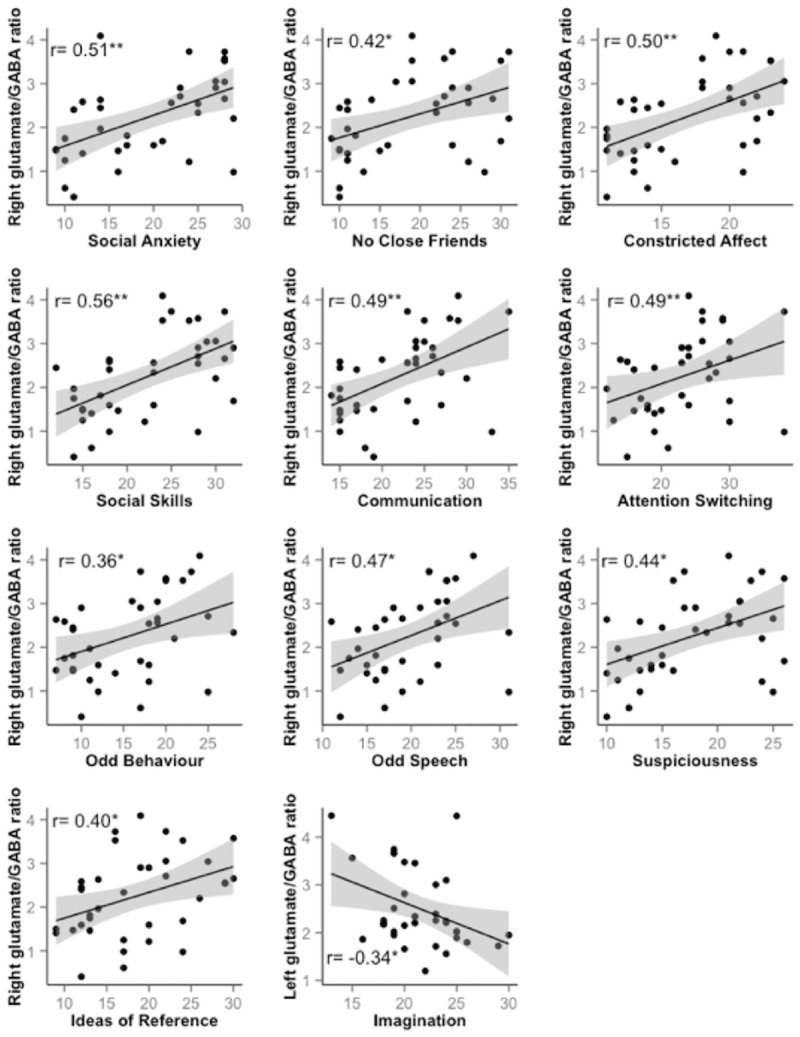
Significant Spearman’s *ρ* correlation coefficients between glutamate/GABA+ ratio and ASQ subscales. Scatter plots of right superior temporal voxel glutamate/GABA+ ratio versus Social Anxiety, No Close Friends, Constricted Affect, Social Skills, Communication, Odd behaviour, Odd Speech, Ideas of Reference and Suspiciousness. *p< 0.05, **Bonferroni corrected p< 0.004.

The Spearman’s *ρ* and adjusted *R*^2^ were then calculated for the relationship between each AQ and SPQ subscale and right and left superior temporal glutamate/GABA+ ratio. There were significant, moderate to strong, positive correlations between glutamate/GABA+ ratio in the right hemisphere voxel and the SPQ subscales Ideas of Reference (*ρ* = 0.4, p = 0.017), Suspiciousness (*ρ* = 0.44, p = 0.007), Social Anxiety (*ρ* = 0.51, p = 0.002), No Close Friends (*ρ* = 0.42, p = 0.01), Constricted Affect (*ρ* = 0.50, p = 0.002), Odd behaviour (*ρ* = 0.36, p = 0.031) and Odd Speech (*ρ* = 0.47, p = 0.004), and between AQ subscales Social Skills (*ρ* = 0.56, p<0.001), Communication (*ρ* = 0.49, p = 0.002) and Attention Switching (*ρ* = 0.38, p = 0.023). There was also a significant increase in left voxel glutamate/GABA+ with lower Imagination scores (*ρ* = -0.34, p = 0.045); no other left hemisphere correlations were significant. A summary of all subscale correlations is presented in Table 3, and Fig 4 illustrates the significant relationships. Of these, the correlations between right glutamate/GABA+ ratio and Social Anxiety, Constricted Affect, Social Skills and Communication survived Bonferroni correction (p<0.004). Correlations between AQ and SPQ subscales and GABA+ and glutamate concentrations are presented in S1 and S2 Tables, respectively.

**Table 3:** Spearman’s *ρ* rank order correlations between right and left superior temporal glutamate/GABA+ ratio and AQ and SPQ subscales.

Hem.	Subscale	Spearman’s *ρ*	Adjusted *R*^2^	p-value
Left	Ideas of reference	0.14	-0.04	0.409
	Odd beliefs	0.30	0.03	0.080
	Unusual perceptual exp.	0.05	-0.06	0.758
	Suspiciousness	0.12	-0.05	0.492
	Social anxiety	-0.12	-0.05	0.507
	No close friends	0.00	-0.06	0.999
	Constricted affect	-0.02	-0.06	0.915
	Odd behaviour	0.02	-0.06	0.913
	Odd speech	0.11	-0.05	0.547
	Social skills	0.00	-0.06	0.992
	Communication	-0.04	-0.06	0.835
	Attention switching	-0.13	-0.05	0.471
	Attention to detail	-0.15	-0.04	0.374
	Imagination	-0.34	0.06	0.045*
Right	Ideas of reference	0.40	0.11	0.017 *
	Odd beliefs	0.06	-0.06	0.742
	Unusual perceptual exp.	0.29	0.03	0.083
	Suspiciousness	0.44	0.15	0.007*
	Social anxiety	0.51	0.21	0.002**
	No close friends	0.42	0.13	0.010 *
	Constricted affect	0.50	0.20	0.002*
	Odd behaviour	0.36	0.08	0.031*
	Odd speech	0.47	0.17	0.004*
	Social skills	0.56	0.28	0.000*
	Communication	0.49	0.19	0.002*
	Attention switching	0.38	0.09	0.023*
	Attention to detail	0.13	-0.04	0.440
	Imagination	0.22	-0.01	0.194

Exp. = Experiences,

*p< 0.05,

**Bonferroni corrected p< 0.004

## Discussion

This study is the first to investigate the relationship between the glutamate/GABA+ ratio in the superior temporal region of the left and right hemisphere and subclinical autistic and schizotypal traits. Following from our previous findings of increased right temporal glutamate/GABA+ ratio [11], the results herein support the hypothesis that right temporal glutamate/GABA+ ratio increases with Social Disorganization-specific AQ and SPQ subscale scores. Increased right superior temporal voxel glutamate/GABA+ ratio was related to an increase in the psychosocial ASQ subscales Social Anxiety, No Close Friends, Constricted Affect, Social Skills, Communication, Odd Speech and Odd Behaviour, as well as for the psychosis-related subscales of Ideas of Reference and Suspiciousness. Importantly, the relationship between glutamate/GABA+ ratio and Social Anxiety, Constricted Affect, Social Skills and Communication scores was robust to multiple comparisons. Conversely, left superior temporal glutamate/GABA+ ratio decreased with more restricted imagination.

Our findings are in line with clinical studies that report increased temporal Glx concentration with more negative symptoms of schizophrenia in patients [28] and increased ACC glutamate/creatine with more negative symptoms and poorer global functioning [27]. Increased left auditory glutamate concentration has also been related to higher AQ scores in those with autism [13], however increasing Glx concentration in the ACC has been related to reduced Communication deficits in those with autism [16]. The relationships between glutamate/GABA+ ratio and the subscales Social Anxiety, Constricted Affect, Social Skills and Communication were the only survivors of multiple comparisons correction, suggesting that perhaps these traits are more closely modulated by the glutamate/GABA+ ratio. Naturally, there are moderate relationships between these subscales [7], and indeed these subscale scores contribute to total AQ, SPQ and ASQ. However, each individual subscale measures slightly different domains of the autism-schizotypy spectra, therefore, the relationship between the glutamate/GABA+ ratio and each subscale, as well as the total scales, is unique and meaningful. Considering the subclinical nature of the participants within this study, these data indicate cortical soft signs of symptom correlates, which may be stronger given a larger sample and increased symptom severity.

GABA is the major regulator of glutamatergic excitation in the cortex, thus a balance of glutamate/GABA is necessary to maintain the balance of excitatory and inhibitory neurotransmitters [25, 26]. Increased glutamate/GABA+ ratio with increased trait expression may indicate that there is an over excitation for those with more social and interpersonal difficulties, and that this relationship is right hemisphere lateralised.

The right cortical hemisphere is responsible for the production, reception and interpretation of prosody, and the paralinguistic aspects of language [46]. Lindell and colleagues [46] provide a comprehensive review of the role of the right hemisphere in language, presenting evidence that a compromised right hemisphere impairs the integration, interpretation and inferences of spoken language. Furthermore, the right hemisphere is important for online interpretation of language, leading to deficits in the understanding of the pragmatics of language when damaged. In sum, the data suggest that the right temporal lobe is essential for social communication, with abnormalities leading deficits in the interpretation of prosody, pragmatics and discourse, which in turn impact one’s ability to interpret another’s intention. Therefore, social and interpersonal deficits may be a downstream outcome of compromised right auditory processes.

Some limitations of this study must be noted. First, sample size of this study is rather small for correlational analyses, therefore these findings are preliminary and warrant further investigation. Furthermore, when inspecting the glutamate/GABA+ ratio and ASQ, AQ and SPQ scale scores for the higher and lower scoring participants separately, the relationship appears to be weak. However, the size of these groups are too small (less than 20) to reject the existence of a relationship. In addition, the correlation analyses were carried out using Spearman’s *ρ*, which accounts for the non-normality in the scales and subscales and the relationships were robust to outliers. These findings provide an important insight into the potential contribution of glutamate and GABA to autistic and schizotypal symptom traits, and add weight to the argument for a shared autism and schizophrenia spectrum phenotype that is comprised of social and interpersonal deficits. Secondly, the voxel sizes were slightly smaller than what is generally recommended in order to focus on the auditory cortices. However, as demonstrated by the spectral images in Fig 2 and the statistical analysis of the MEGA-PRESS and 80TE PRESS fit parameters, the GABA+ and glutamate concentrations are reliable. The findings in this study provide preliminary evidence for an association between autistic and schizotypal traits and glutamate/GABA+ ratio, with larger studies required to confirm this association.

## Conclusion

This study is the first to investigate glutamate/GABA+ ratio in autistic and schizotypal traits in a high functioning population. The findings illustrate that subclinical glutamate/GABA+ ratio is related to the degree of social and interpersonal difficulties shared within the autism and schizophrenia spectra. These data support the emergence of the Social Disorganization phenotype and provide preliminary steps into the understanding of excitation/inhibition in social and interpersonal functioning, which adds to the growing literature regarding the relationship between autism and schizophrenia spectrum disorders.

## Supporting information

S1 Table Correlations between right and left superior temporal GABA+ concentration and AQ and SPQ subscales. Table of correlations between right and left superior temporal GABA+ concentration and AQ and SPQ totals and subscales.
(PDF)

S2 Table Correlations between right and left superior temporal glutamate concentration and AQ and SPQ subscales. Table of correlations between right and left superior temporal glutamate concentration and AQ and SPQ totals and subscales.
(PDF)
