# Supplementary material for: Glutamate/GABA+ ratio is associated with the psychosocial domain of autistic and schizotypal traits
Source: PLoS One. 2017 Jul 31;12(7):e0181961. doi: 10.1371/journal.pone.0181961 (PMC5536272; doi:10.1371/journal.pone.0181961)
Supplement: S1 Table — Table of correlations between right and left superior temporal GABA+ concentration and AQ and SPQ totals and subscales. (PDF) [file pone.0181961.s001.pdf]

S1 Table

Glutamate/GABA+ Ratio is associated with the Psychosocial Domain of Autistic and Schizotypal Traits

Talitha C. Ford, Richard Nibbs and David P. Crewther

Table 1. Correlations between right and left superior temporal GABA+ concentration and AQ and SPQ subscales.

Hem.	Subscale	Pearson's r	Adjusted R^2	p-value
Left	Ideas of Reference	0.28	0.05	0.103
	Odd Beliefs	-0.14	-0.01	0.435
	Unusual Perceptual Exp.	0.18	0.00	0.289
	Suspiciousness	0.33	0.08	0.055
	Social Anxiety	0.40	0.13	0.018
	No Close Friends	0.24	0.03	0.162
	Constricted Affect	0.26	0.04	0.125
	Odd Behaviour	0.28	0.05	0.100
	Odd Speech	0.19	0.01	0.281
	SPQ Total	0.30	0.06	0.079
	Social Skills	0.28	0.05	0.102
	Communication	0.24	0.03	0.167
	Attention Switching	0.38	0.12	0.025
	Attention To Detail	0.06	-0.03	0.729
	Imagination	0.37	0.11	0.029
	AQ Total	0.34	0.09	0.045
	ASQ Total	0.32	0.08	0.059
Right	Ideas of Reference	-0.18	0.01	0.284
	Odd Beliefs	-0.15	-0.01	0.393
	Unusual Perceptual Exp.	-0.11	-0.02	0.513
	Suspiciousness	-0.33	0.08	0.052
	Social Anxiety	-0.36	0.10	0.031
	No Close Friends	-0.30	0.06	0.080
	Constricted Affect	-0.37	0.11	0.028
	Odd Behaviour	-0.20	0.01	0.243
	Odd Speech	-0.25	0.03	0.144
	SPQ Total	-0.31	0.07	0.069
	Social Skills	-0.39	0.12	0.020
	Communication	-0.25	0.04	0.139
	Attention Switching	-0.21	0.01	0.225
	Attention To Detail	-0.11	-0.02	0.524
	Imagination	-0.22	0.02	0.197
	AQ Total	-0.30	0.06	0.075
	ASQ Total	-0.31	0.07	0.065

AQ= autism spectrum quotient, SPQ= schizotypal personality questionnaire, ASQ= autism schizotypy questionnaire
