# Supplementary material for: Glutamate/GABA+ ratio is associated with the psychosocial domain of autistic and schizotypal traits
Source: PLoS One. 2017 Jul 31;12(7):e0181961. doi: 10.1371/journal.pone.0181961 (PMC5536272; doi:10.1371/journal.pone.0181961)
Supplement: S2 Table — Table of correlations between right and left superior temporal glutamate concentration and AQ and SPQ totals and subscales. (PDF) [file pone.0181961.s002.pdf]

S2 Table

Glutamate/GABA+ Ratio is associated with the Psychosocial Domain of Autistic and Schizotypal Traits

Talitha C. Ford, Richard Nibbs and David P. Crewther

Table 1. Correlations between right and left superior temporal glutamate concentration and AQ and SPQ subscales.

Hem.	Subscale	Pearson's r	Adjusted R^2	p-value
Left	Ideas of Reference	0.48	0.21	0.003
	Odd Beliefs	0.32	0.08	0.054
	Unusual Perceptual Exp.	0.38	0.12	0.022
	Suspiciousness	0.49	0.22	0.002
	Social Anxiety	0.33	0.08	0.048
	No Close Friends	0.31	0.07	0.065
	Constricted Affect	0.29	0.06	0.084
	Odd Behaviour	0.25	0.03	0.150
	Odd Speech	0.34	0.09	0.043
	SPQ Total	0.42	0.15	0.011
	Social Skills	0.31	0.07	0.067
	Communication	0.21	0.02	0.223
	Attention Switching	0.19	0.01	0.268
	Attention To Detail	-0.04	-0.03	0.809
	Imagination	-0.03	-0.03	0.866
	AQ Total	0.20	0.01	0.249
	ASQ Total	0.35	0.10	0.035
Right	Ideas of Reference	0.25	0.03	0.146
	Odd Beliefs	-0.03	-0.03	0.847
	Unusual Perceptual Exp.	0.10	-0.02	0.543
	Suspiciousness	0.23	0.02	0.178
	Social Anxiety	0.33	0.08	0.049
	No Close Friends	0.33	0.08	0.047
	Constricted Affect	0.34	0.09	0.042
	Odd Behaviour	0.06	-0.03	0.728
	Odd Speech	0.23	0.02	0.181
	SPQ Total	0.27	0.05	0.113
	Social Skills	0.30	0.06	0.079
	Communication	0.27	0.04	0.117
	Attention Switching	0.20	0.01	0.246
	Attention To Detail	-0.06	-0.03	0.709
	Imagination	0.05	-0.03	0.769
	AQ Total	0.22	0.02	0.193
	ASQ Total	0.26	0.04	0.129

AQ= autism spectrum quotient, SPQ= schizotypal personality questionnaire, ASQ= autism schizotypy questionnaire
